# Erythropoietin Down-Regulates Stem Cell Factor Receptor (Kit) Expression in the Leukemic Proerythroblast: Role of Lyn Kinase

**DOI:** 10.1371/journal.pone.0005721

**Published:** 2009-05-28

**Authors:** Olivier Kosmider, Dorothée Buet, Isabelle Gallais, Nicole Denis, Françoise Moreau-Gachelin

**Affiliations:** 1 Inserm U830, Paris, France; 2 Institut Curie, Paris, France; Roswell Park Cancer Institute, United States of America

## Abstract

Overexpression of the transcription factor Spi-1/PU.1 by transgenesis in mice induces a maturation arrest at the proerythroblastic stage of differentiation. We have previously isolated a panel of *spi-1* transgenic erythroleukemic cell lines that proliferated in the presence of either erythropoietin (Epo) or stem cell factor (SCF). Using these cell lines, we observed that EpoR stimulation by Epo down-regulated expression of the SCF receptor Kit and induced expression of the Src kinase Lyn. Furthermore, enforced expression of Lyn in the cell lines increased cell proliferation in response to Epo, but reduced cell growth in response to SCF in accordance with Lyn ability to down-regulate Kit expression. Together, the data suggest that Epo-R/Lyn signaling pathway is essential for extinction of SCF signaling leading the proerythroblast to strict Epo dependency. These results highlight a new role for Lyn as an effector of EpoR in controlling Kit expression. They suggest that Lyn may play a central role in during erythroid differentiation at the switch between proliferation and maturation.

## Introduction

Erythropoiesis is critically regulated by a number of growth factors acting through specific receptors, among which erythropoietin (Epo) and stem cell factor (SCF) are essential factors [1]. SCF, the ligand for the Kit receptor, is mainly involved in the survival and proliferation of immature erythroid progenitors, whereas Epo is the predominant regulator preventing apoptosis at the CFU-E/proerythroblast stage of differentiation. The importance of the SCF/Kit pathway during erythropoiesis was highlighted in mice with inactivating mutation in the SCF (Sl/Sl mice) or Kit gene (W/W mice) [2], [3]. Mutant mice die *in utero* between day 14–16 of gestation with anemia and a profoundly reduced number of erythroid progenitors in fetal liver demonstrating the proliferative function mediated by Kit during early stages of erythropoiesis. Likewise, mice with null mutations in the genes encoding either Epo or EpoR die at midgestation with a severe anemia. Fetal livers from these mice contain BFU-E and CFU-E progenitors, although in reduced number, indicating that the Epo/EpoR pathway is crucial in regulating survival, proliferation and terminal differentiation of CFU-E [4]. Thus, Epo and SCF are growth factors working synergistically to support erythropoiesis, with SCF exerting a predominant role to expand early progenitors, while Epo is acting later on to sustain maturation.

Signaling induced by Epo/EpoR and SCF/Kit is determined by the temporal and spatial expression of their cognate receptors at the surface of responsive cells. Kit is expressed from the earliest committed erythroid progenitor up to the basophilic erythroblastic stage of differentiation [5], [6]. EpoR expression arises at the BFU-E stage, reaches a maximum at the CFU-E and proerythroblast stages and declines thereafter [7], [8].

In an attempt to dissect the signaling determinants controlling the expression of EpoR and Kit, we used proerythroblastic cell lines isolated during the preleukemic step of erythroleukemia developing in *spi-1* transgenic mice [9]. The *spi-1* gene encodes the ETS transcription factor Spi-1/PU.1, a main player regulating the commitment of multipotent hematopoietic progenitors and the development of the B lymphoid and monocytic lineages [10]–[13]. Germline overexpression of the *spi-1* transgene induces a differentiation arrest in the erythroid lineage at the CFU-E/proerythroblast transition leading to severe anemia [9], [14]. In response to anemia, Epo production is up-regulated [15] causing a massive expansion of proerythroblasts in the hematopoietic tissues of diseased mice. It is likely that SCF expressed by stromal cells in spleen and marrow microenvironments also contributes to the expansion of these proerythroblasts. Indeed, *spi-1* transgenic proerythroblasts express both Epo and SCF receptors and can be expanded *in vitro* in the presence of Epo or SCF.

Using cell lines established from the spleen of various diseased mice, we observed that each of these cell lines exhibited a particular growth rate in response to either Epo alone or SCF alone, and expressed EpoR and Kit in a ratio modulated by the cytokine used to sustain their proliferation. Starting from this observation, we investigated the molecular mechanisms controlling the expression of Kit and EpoR. We show that Epo down-regulated Kit expression and induced expression of the Lyn kinase. When ectopically expressed in *spi*-transgenic proerythroblasts, Lyn favored cell proliferation in response to Epo, but not to SCF. These biological effects are consistent with the ability of Lyn to induce a down-regulation of Kit expression. Our findings reveal a novel aspect of signaling crosstalk between Kit and EpoR and highlight a central role for Lyn in SCF signaling extinction at the CFU-E/proerythroblast stage.

## Materials and Methods

### Cell lines and proliferation assays

The *spi-1*-transgenic proerythroblastic cell lines (633, 663, 812) have been previously described in details [9]. Cells were grown in alpha MEM medium supplemented with 10% fetal bovine serum (FBS) and SCF or Epo or both cytokines in combination at the indicated concentrations. Cells were plated at 2×10^5^ cells/mL and the number of living cells was monitored at 48 hours by Trypan blue exclusion using a Vi-Cell analyzer (Beckman Coulter, Villepinte, France). For cytokine switching experiments, exponentially growing cells were washed 3 times in MEM and then plated at 2×10^5^ cells/mL in culture medium with the cytokines indicated. AG490 (Calbiochem, Strasbourg, France) and JAK inhibitor1 (Calbiochem, Strasbourg, France) were used at a concentration of 10 µM and 20 nM, respectively.

### Flow cytometry and antibodies

Untreated cells were incubated for 30 min at 4°C with Phycoerythrin (PE)-conjugated anti-CD117 (c-Kit) or PE-conjugated IgG_2b_ control monoclonal antibodies (BD Pharmingen, Strasbourg, France). After washing, cells were analyzed on a FACsort® with the Cellquest software package (Becton Dickinson, Meylan, France).

### DNA constructs and transfection

The wild-type (WT) *Lyn* cDNA was amplified by RT-PCR from mRNAs prepared from 663 cells. The Myc epitope (MT) was added at the cDNA C-terminus by PCR and the *MT-Lyn^WT^* construct was cloned into the pEF-BOS expression vector by standard cloning procedures. The mutant *Lyn^Y397F^* cDNA was generated by mutagenesis of the *MT-Lyn^WT^* construct using the quickchange site-directed mutagenesis system (Stratagene, La Jolla, CA) according to the manufacturer's recommendations. Cells were nucleofected with 5 µg of plasmid using an Amaxa nucleofector (Amaxa Biosystems, Köln, Germany). Stable transfectants were selected in growth medium containing 800 µg/mL G418 (Invitrogen, Cergy, France) and Epo (1 U/mL).

### Western blotting and antibodies

Whole cell extracts were fractionated by SDS-PAGE, blotted and visualized as previously described [16]. The following primary antibodies were used: rabbit anti-Kit antibodies provided by P. Dubreuil (Inserm, Marseille, France) [17] or from Cell Signaling (Beverly, MA), anti-EpoR, anti-Lyn and anti-Stat5 antibodies from Santa Cruz Biotechnology (Santa Cruz, California), anti-phosphotyrosine 4G10 clone from Upstate Biotechnology (Lake Placid, NY), anti-phospho-c-Kit (Tyr719) and anti-phospho-Stat5 (Tyr694) from Cell Signaling (Beverly, MA), anti-β actin antibody from Sigma-Aldrich (St Louis, MO) and anti-myc (epitope 9E10) from Roche Diagnostics (Mississauga, Ontario, Canada).

### Semiquantitative RT-PCR

RNAs were prepared as previously descibed [9]. RNAs were reverse transcribed using Superscript II reverse transcriptase (Invitrogen). cDNAs were amplified by RT-PCR with specific primers for *Kit*, *EpoR* and *Lyn*: *Kit*-fw5′-TCCTCgCCTCCAAgAATTg-3′ and *Kit*-rev 5′-ggAAgCCTTCCTTgATCATC-3′, *EpoR*-fw 5′- ggCTCCgAAgAAcTTCTgTg-3′ and *EpoR*-rev 5′- CCAggAgCACTACTTCATTg-3′, *Lyn*-fw 5′-GATCCAGAGGAACAAGGTGA-3′ and *Lyn*-rev 5′-TGACATCACCATGCATAGGG-3′.

## Results

### Kit expression is modulated by cytokines

**Figure 1:**
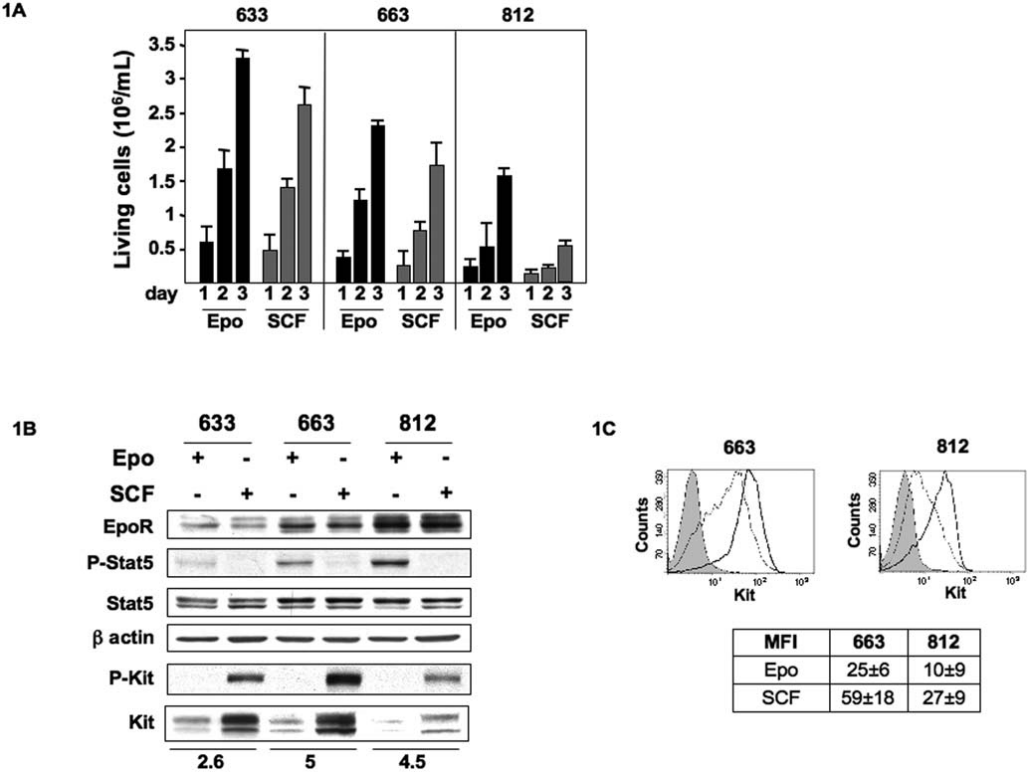
Proliferation of *spi-1* transgenic proerythroblasts and expression of EpoR, Kit and Stat5. A: Cells were continuously cultured in the presence of Epo (1 U/mL) or SCF (100 ng/mL). Number of living cells was monitored at 24, 48 and 72 hours using the Trypan blue exclusion staining and a Vi-Cell analyzer (Becton Coulter). Mean number of living cells and standard deviations were determined from 3 independent experiments performed in duplicate. B: Representative Western blot of lysates from cells grown with Epo (1 U/mL) or SCF (100 ng/mL). Antibodies raised against the proteins are indicated on the left of the panel. P-Stat5 and P-Kit antibodies detect Stat5 and Kit phosphorylated forms. The blot was probed with an anti-β-actin antibody to visualize the protein loading. The membrane was exposed in an Imager, and the resulting signal was quantified using the ImageGauge software package (Fuji, Paris, France). Values were normalized to β actin expression. The fold change in Kit expression between Epo or SCF-cultured cells is indicated at the bottom of each cell line. C: Representative diagram of flow cytometry analysis showing cell surface expression of Kit in 663 and 812 cells cultured with Epo (dotted line) or SCF (black line). Control IgG profile is shown in grey. The table indicates the mean fluorescence intensity (MFI)±SD of positive cells from four independent experiments.

The three proerythroblastic cell lines (633, 663, 812) used in this study were derived from erythroleukemic spleens of three individual *spi-1* transgenic mice [9]. The cells were continously amplified *in vitro* in the presence of either Epo (1 U/mL) or SCF (100 ng/mL) [18]. Each cell line exhibited characteristic proliferation rates that were reproducibly observed over times. In response to either Epo or SCF, 633 cells were highly proliferative, 663 cells showed an intermediary proliferation rate and 812 cells proliferated at a low rate (Figure 1A). To gain insights into the possible causes leading to proliferation rate disparity, we analyzed the expression level of receptors for Epo (EpoR) and SCF (Kit) by immunoblotting. Different levels of EpoR expression were detected in each cell line, but these levels were comparable whether the cells were cultured with Epo or SCF (Figure 1B). Unexpectedly, the highest EpoR levels were seen in 812 cells that proliferated poorly in response to Epo. To check for EpoR activity, we analyzed Stat5 phosphorylation [19], [20]. Phosphorylated Stat5 (P-Stat5) was detected in the 3 cell lines grown with Epo with the highest level seen in 812 cells that exhibited the highest EpoR expression level. P-Stat5 was undetectable in cells grown with SCF (Figure 1B). Next, we analyzed Kit expression levels in the 3 cell lines grown under the 2 cytokines conditions. Clearly, Kit levels were high in cells exhibiting a robust proliferative response to SCF (cell lines 633 and 663). As a read out for Kit activity, detection of the phosphorylated-Y719-Kit form was performed by immunoblotting. P-Y719-Kit was clearly seen in all cells grown with SCF, but not in Epo-cultured cells (Figure 1B). Strikingly, total Kit expression levels were significantly higher in cells grown with SCF compared to cells grown with Epo. Modulation in Kit expression was also detected at the surface of cells grown with Epo or SCF by flow cytometry analysis. The mean Kit-specific fluorescence (MFI) level was increased about 2.5 fold in 663 and 812 cells cultured with SCF compared to cells grown with Epo (Figure 1C). Similar results were obtained for 633 cells (data not shown). Collectively, these data suggested that cytokines could modulate Kit expression levels in leukemic proerythroblastic cells, while no major effect were seen on Epo-R expression.

### Kit expression is under Epo control

**Figure 2:**
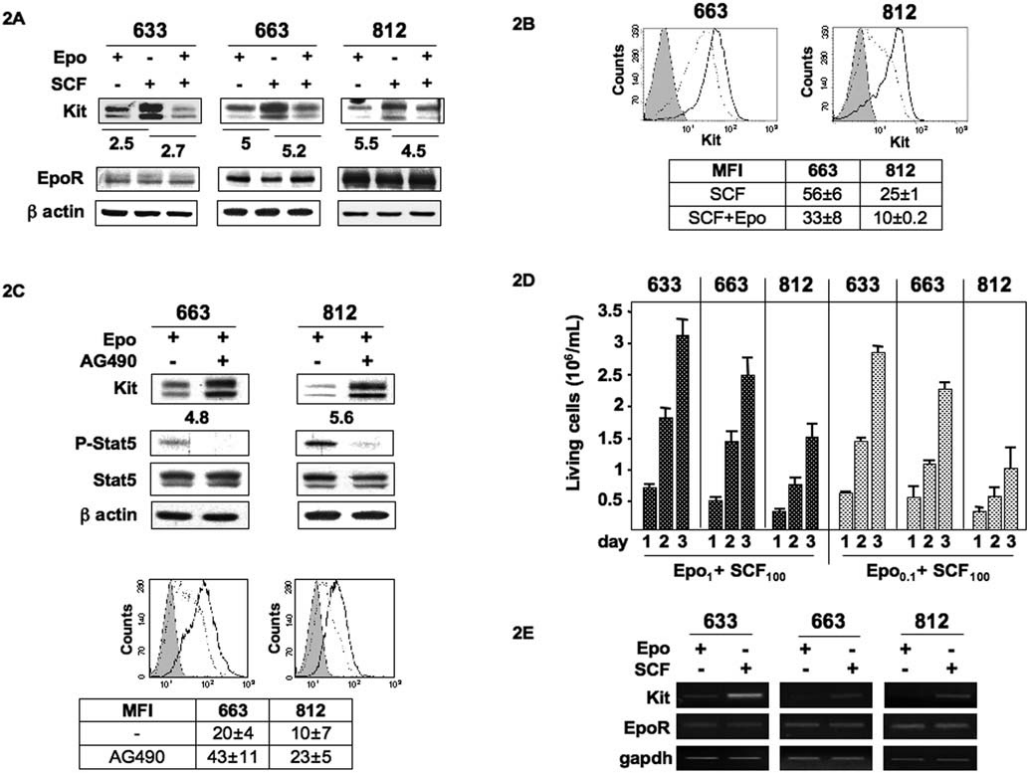
Down-regulation of Kit expression by Epo. A: Kit and EpoR expression were studied in the 633, 663 and 812 cells grown continuously in the presence of either Epo (1 U/mL) or SCF (100 ng/mL) or a combination of Epo (1 U/mL)+SCF (100 ng/mL). Whole cell lysates were subjected to Western blot analysis with antibodies directed against Kit, EpoR and β-actin as a loading control. Western blots are from a representative experiment. The membrane was exposed in an Imager, and the resulting signal was quantified using the ImageGauge software package (Fuji, Paris, France). Values were normalized to β actin expression. The fold change in Kit expression between SCF-cultured cells and either Epo or Epo+SCF-cultured cells is indicated under Kit immunoblotting. B: Representative diagram of flow cytometry analysis showing Kit membrane expression in cells cultured with SCF (100 ng/mL; black line) or Epo+SCF (dotted line). Control IgG profile is shown in grey. The table indicates the mean fluorescence intensity (MFI)±SD of four independent experiments. C: AG490 inhibits the down-regulation of Kit by Epo. Cells were cultured for 48 hrs in a medium containing 10% FBS, 1 U/mL Epo and in the presence or absence (-) of AG490 (10 µM). Representative Western blot analysis of whole cell lysates with antibodies directed against Kit, Stat5 and phosphorylated Stat5. β-actin was used as loading control. The fold change in Kit expression between AG490-treated and untreated cells is indicated under Kit immunoblotting. Representative diagram of flow cytometry analysis showing Kit membrane expression in 663 and 812 cells cultured with Epo and treated (black line) or not (dotted line) with AG490 (10 µM) for 48 hrs. Control IgG profile is shown in grey. The table indicates the mean fluorescence intensity (MFI)±SD of positive cells in three independent experiments. D: Cells were cultured for 24, 48 and 72 hrs in the presence of a combination of Epo (1 U/mL)+SCF (100 ng/mL) or Epo (0.1 U/mL)+SCF (100 ng/mL) and viable cells were numbered. Data are mean±SD of five experiments, each performed in duplicate. E: The regulation of Kit expression is transcriptional: RT-PCR analysis of *Kit* and *EpoR* transcripts in 633, 663 and 812 cells cultured in the presence of either Epo (1 U/mL) or SCF (100 ng/mL). cDNAs were amplified with specific primers for *Kit*, *EpoR* or *gapdh* as control.

Next, we compared the EpoR and Kit expression levels in the cell lines cultured in a combination of Epo+SCF (SCF was added in Epo-cultured cells for 5 days) to cells cultured with either Epo alone or SCF alone. On Western blotting, Kit expression levels were high with SCF alone, but significantly reduced when Epo was present (Figure 2A). Similarly, cell surface modulation of Kit expression was also detected by flow cytometry analysis with a Kit-specific MFI higher in cells grown with SCF alone than in cells expanded with Epo+SCF (Figure 2B). These data were evocative of a modulation of Kit expression by Epo. To check this hypothesis, we investigated the effects of AG490 and JAK inhibitor 1, both inhibiting Jak2 kinase activity. P-Stat5 was used as a read out for the inhibitory effects of AG490 and JAK inhibitor 1. Both inhibitors had similar effects and only data with AG490 were shown. After a 48 hrs exposure to AG490 (10 µM), P-Stat5 was undetectable on Western blotting in cells cultured with Epo (1 U/mL), although total Stat5 levels were unchanged (Figure 2C). Strikingly, inhibition of EpoR signaling in AG490-treated cells was accompanied by an increase in Kit expression as compared to untreated cells. The Kit increase was also detected at the cell surface of AG490-treated cells by flow cytometry analysis (Figure 2B). Altogether, these data indicated that the modulation of Kit expression was dependent on Jak2 activity and suggested that activation of EpoR could act as a repressor of Kit expression.

We next assessed whether the modulation of Kit expression by Epo affected the proliferation of cells cultured in the presence of a combination of Epo+SCF (Figure 2D). When Epo (1 U/mL) was combined with SCF (100 ng/mL) cell growth was similar to that in the presence of Epo (1 U/mL) alone (Figure 1A), indicating that Epo and SCF did not cooperate for cell proliferation. In contrast, a cooperative effect on proliferation was observed when SCF (100 ng/mL) was combined to Epo at a limiting dilution (0.1 U/mL) (Figure 2D) since cell growth was comparable to Epo alone (1 U/mL) (Figure 1A). These data indicated that the low expression of Kit was the reason why Epo at high dose did not cooperate with SCF.

To learn more about the molecular mechanisms involved in the Epo-dependent regulation of Kit protein expression, we first investigated whether this process was associated to variations in *Kit* transcription. The expression level of *kit* transcripts was analyzed by semi-quantitative RT-PCR using RNAs extracted from cells cultured in the presence of either Epo or SCF (Figure 2E). The expression level of *EpoR* transcripts was similarly investigated. Amplification of *Kit* transcripts was elevated in SCF-cultured cells but was poorly efficient in Epo-cultured cells whereas *EpoR* transcript levels were not affected by cytokines. These data strongly suggested that the regulation of Kit expression by Epo was dependent on a transcriptional process.

### Reversible down-regulation of Kit in response to Epo

**Figure 3:**
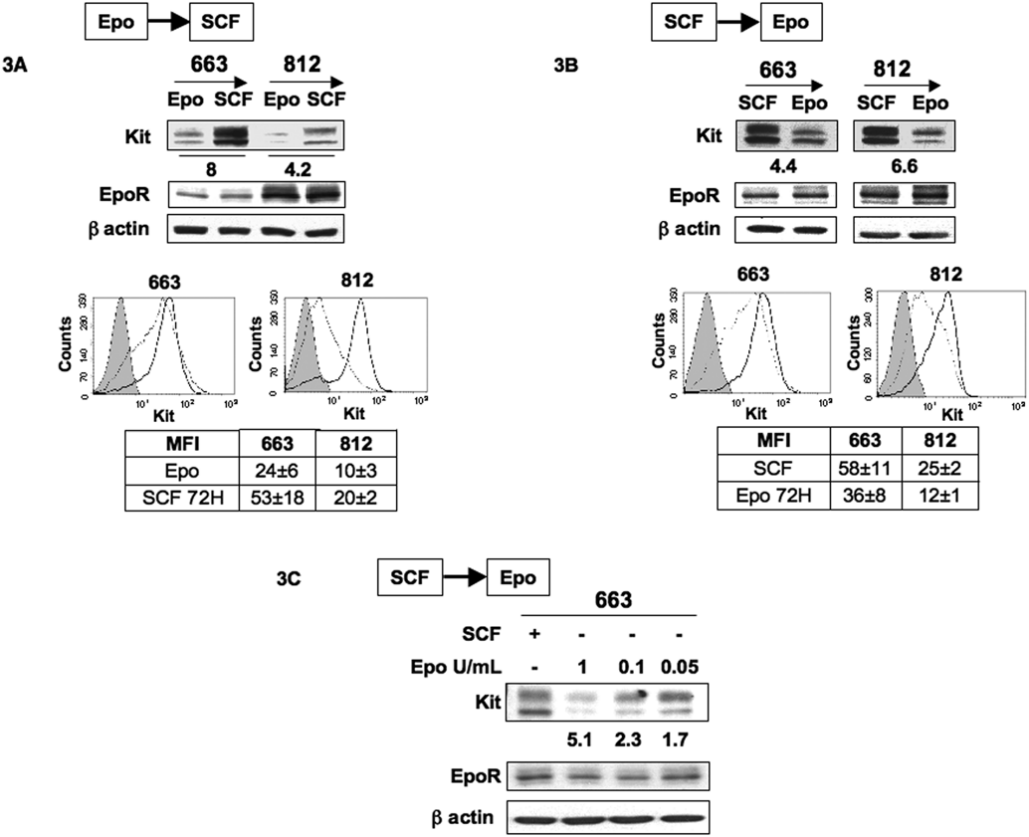
Reversible down-regulation of Kit in response to Epo. Switch from Epo to SCF (A) and switch from SCF to Epo (B): 663 and 812 cells continuously cultured with Epo (1 U/mL) or SCF (100 ng/mL) were extensively washed with medium without cytokine and then expanded for 48 hrs with SCF (100 ng/mL) or Epo (1 U/mL), respectively. Whole cell lysates were subjected to Western blot analysis with antibodies directed against Kit, EpoR and β-actin as a loading control. The fold change in Kit expression following the switch in cytokines is indicated under Kit immunoblotting. Representative diagrams of flow cytometry analysis showing Kit expression on the surface of cells expanded with Epo (dotted line) or SCF (black line). Control IgG profile is shown in grey. The tables indicate the mean fluorescence intensity (MFI)±SD of positive cells in at least four independent experiments. C: Switch from SCF to various concentrations of Epo. 663 cells cultured with SCF (100 ng/mL) were extensively washed with medium without cytokine and then expanded for 48 hrs with Epo at doses indicated. Whole cell lysates were subjected to Western blot analysis with antibodies directed against Kit, EpoR and β-actin as a loading control. The fold decrease in Kit expression following the switch from SCF to various doses of Epo is indicated under Kit immunoblotting. Western blots are from a representative experiment repeated 3 times.

The above data were obtained with cells continously cultured with Epo or SCF. Thus, we cannot exclude that they reflected the abilities of Epo and SCF to favor some cell selection rather than a genuine process of response to cytokines. Thus, we investigated whether Kit levels could be reversibly modulated in response to a change in cytokines. 663 and 812 cells cultured in the continuous presence of Epo (1 U/mL) were extensively washed and then switched to SCF (100 ng/mL). 48 hrs later, cell extracts were analyzed by immunobloting with an anti-Kit antibody. Switching from Epo to SCF induced a marked increase in Kit level (Figure 3A). Reciprocally, Kit levels were decreased when cells were switched from SCF to Epo for 48 hrs (Figure 3B). These variations in Kit expression were also detected at the cell surface by flow cytometry analysis. The Kit-specific MFI was increased when cells were switched from Epo to SCF and conversely was decreased after switching from SCF to Epo (Figures 3A, 3B). When cells continuously grown with SCF were switched to various concentrations of Epo for 48 hrs, Kit expression levels were reduced in an Epo-dose dependent manner (Figure 3C). The rapid up or down modulation of Kit levels in response to cytokine changes, the absence of cell death (data not shown) during cytokine changes and the reversibility of the phenomenon indicated that they did not result from a selection process. We thus concluded that activation of Epo-R could mediate a fine regulation on Kit expression.

### Lyn is a downstream Epo-R effector expressed in response to Epo

**Figure 4:**
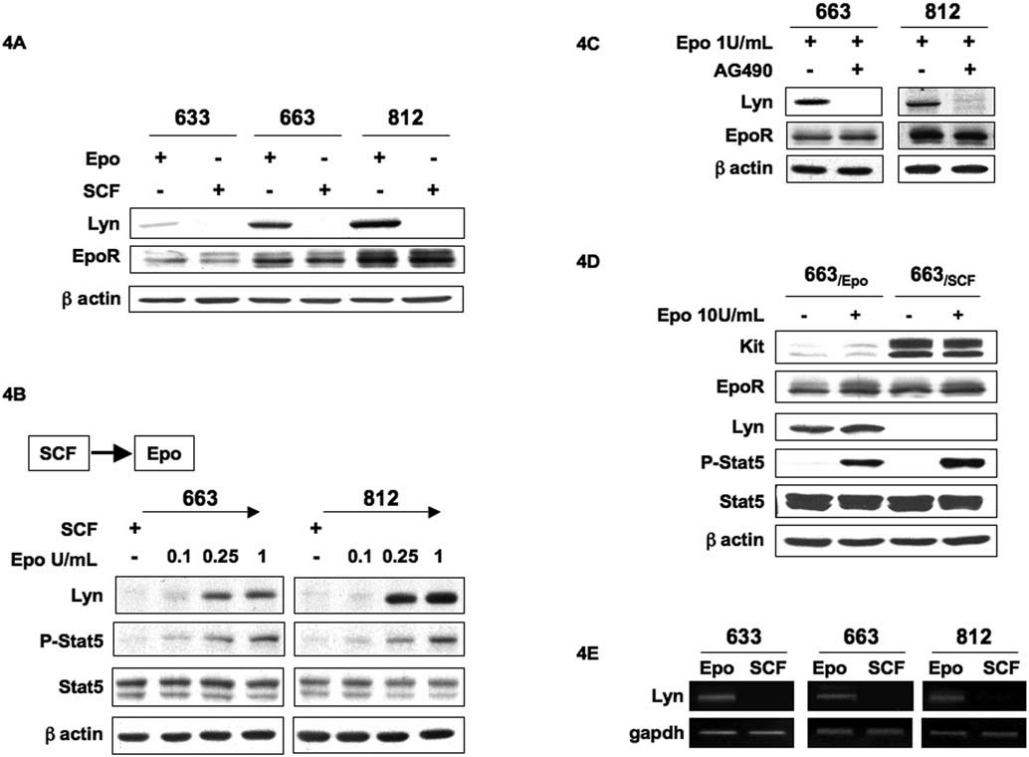
Epo controls Lyn expression. A: Lyn and EpoR expression were studied in 633, 663 and 812 cells continuously grown in the presence of either Epo (1 U/mL) or SCF (100 ng/mL). Whole cell lysates were subjected to Western blot analysis with antibodies directed against Lyn, EpoR and β-actin as a loading control. B: Switch from SCF to Epo induces the expression of Lyn. 663 and 812 cells cultured with SCF (100 ng/mL) were extensively washed with medium without cytokine and then expanded for 48 hrs with Epo at the indicated doses. Whole cell lysates were subjected to Western blot analysis with antibodies directed against Lyn, Stat5, phosphorylated-Stat5 and β-actin as loading control. C: AG490 inhibits the expression of Lyn. 663 and 812 cells were cultured for 48 hrs in a medium containing 10% serum in the presence or absence of AG490 (10 µM) and in the presence of Epo (1 U/ml). Whole cell lysates were subjected to Western blot analysis with antibodies to Lyn, EpoR and β-actin. Western blots are from a representative experiment. D: RT-PCR analysis of *Lyn* transcription in 633, 663 and 812 cells cultured in the presence of either Epo (1 U/mL) or SCF (100 ng/mL). DNAs were amplified with specific primers for *Lyn* or *gapdh* as control.

The next issue was to identify the effectors downstream of EpoR that may participate in the regulation of Kit expression. According to the known function of Src kinases in signaling from growth factors receptors [21], we investigated the expression of Src kinases in 633, 663 and 812 cells when cultured either in the presence of Epo or SCF. The expression of Lyn, Lck and Src could be detected in these cells. We found that Lyn expression was different according to the cytokines added in the culture medium. Immunoblotting with the anti-Lyn antibody in Figure 4A showed that Lyn was expressed in cells cultured with Epo but not with SCF. In contrast, no differences in Lck and Src expressions were observed that cells were cultured with either Epo or SCF (data not shown). Of note, Lyn expression profiles differed in each cell lines and correlated with EpoR expression levels. Thus, we investigated whether Epo could regulate Lyn expression. SCF-cultured cells were extensively washed in culture medium with no cytokine and then cultured for 48 hrs in the presence of increasing Epo concentrations. Immunoblotting of cell extracts with the anti-lyn antibody revealed that Lyn expression was induced in a dose dependent manner with levels readily detectable at 0.25 U/mL of Epo (Figure 4B). Likewise, P-Stat5 activation levels increased with Epo concentration. Thus, Epo induced both Lyn expression and Stat5 activation in a dose-dependent manner. To conclusively confirm the role of EpoR activation in the modulation of Lyn expression, the effect of AG490 on Lyn expression level was investigated by Western blotting (Figure 4C). Lyn expression was abolished when AG490 (10 µM) was added in the culture medium containing Epo, which indicated that Lyn expression was dependent on EpoR/Jak2 activation. Together, these data demonstrated that Lyn expression is under Epo control in *spi-1* transgenic proerythroblasts.

To determine whether the variations in Lyn protein expression were associated to variations in the expression level of *Lyn* transcripts, RT-PCR analyses of RNAs prepared from cells continuously cultured with either Epo or SCF were performed. *Lyn* transcripts were only detected in Epo-cultured cells (Figure 4E) suggesting that Epo might transcriptionally control *Lyn* expression.

### Lyn down-regulates Kit expression

**Figure 5:**
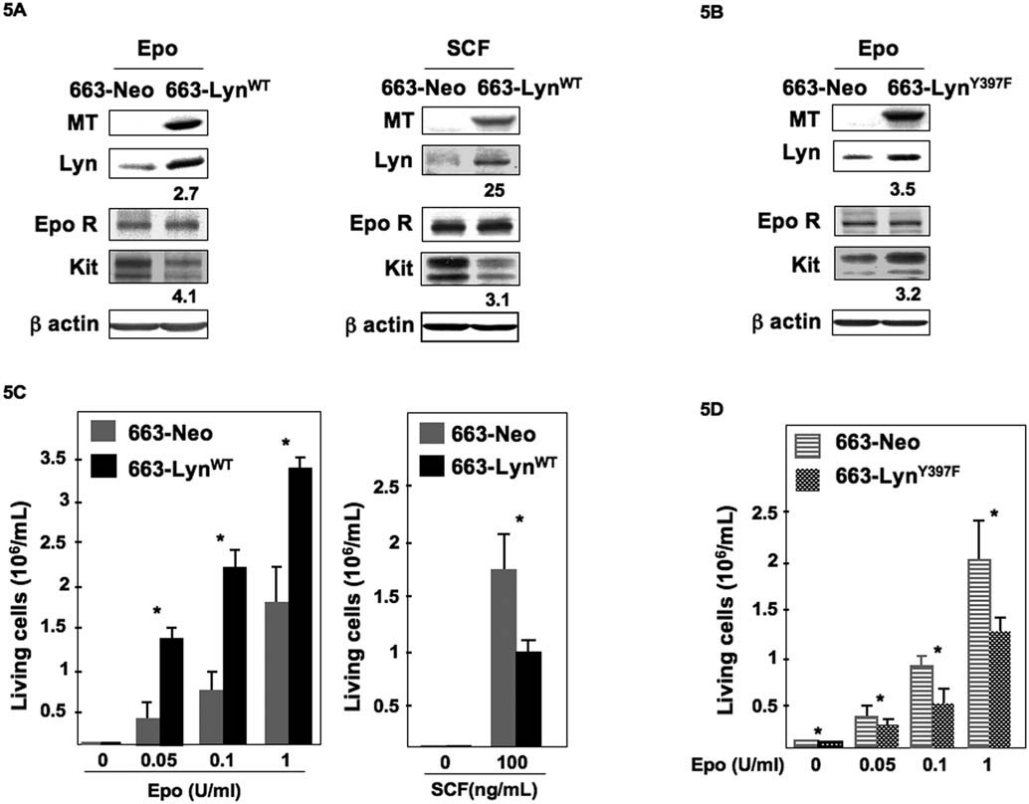
Expression of Lyn^WT^ and Lyn^Y397F^ in 663 cells. (A) A clone of 663 cells stably transfected with pEF-neo Lyn^WT^ or pEF-Neo empty vector was expanded in the presence of Epo (1 U/mL) or SCF (100 ng/mL). Whole cell extracts were subjected to Western blot using anti-MT, anti-Lyn, anti-EpoR, anti-Kit antibodies and anti-β actin as a loading control. The fold increase in Lyn expression and the fold decrease in Kit expression between pEF-Neo and pEF-neo Lyn^WT^ transfected cells are indicated under Lyn or Kit immunoblotting. (B) 663 cells were transfected with pEF-neo Lyn^Y397F^ or pEF-Neo empty vector. Proliferation of 663 cells expressing Lyn^Y397F^ and cultured in the presence of Epo (1 U/mL). Whole cell extracts were subjected to Western blot using an anti-MT, anti-Lyn, an EpoR, an anti-Kit and an anti-β actin antibody as a loading control. The fold increases in Lyn and Kit expressions between pEF-Neo and pEF-neo Lyn^Y397F^ transfected cells are indicated under Lyn or Kit immunoblotting. C: Transfected 663-neo and 663-Lyn^WT^ cells were plated at 2×10^5^ cells/mL in medium containing Epo or SCF at concentrations indicated or with no cytokine. Viable cells were scored after 48 hours. Data are mean±SD of five independent experiments performed in triplicate. *indicates statistical significance by student *t* test: P<.05 compared with the control neo-cells. D: Transfected 663-neo and 663-Lyn^Y397F^ cells were plated at 2×10^5^ cells/mL in the presence of increasing concentrations of Epo and viable cells were scored after 48 hours. Data are mean±SD of five independent experiments performed in triplicate. Results are shown for one 663-Lyn^WT^ clone and one 663-Lyn^Y397F^ clone. Experiments were performed with another clone in each category and similar results were obtained. *indicates statistical significance by student *t* test: P<.05 compared with the control neo-cells.

To explore whether Lyn was a link between EpoR activation and Kit down-regulation, we used an enforced expression strategy. An expression vector encoding Lyn tagged with a Myc epitope (MT) at the C-terminus (MT-Lyn^WT^) and the neomycine resistance gene (NeoR) was stably transfected in Epo-cultured 663 cells. Control cells were transfected with an empty vector encoding NeoR. G418-selected clones were selected in the presence of Epo and then amplified in the presence of Epo or SCF. Among those, two MT-Lyn^WT^ and two control clones were studied in details and gave similar results. Only data with one clone are shown herein. Expression of exogenous MT-Lyn^WT^ was detected by Western blotting with an anti-MT antibody and Lyn global expression was measured with an anti-Lyn antibody (Figure 5A). Whether transfected cells were cultured with Epo (1 U/mL) or SCF (100 ng/mL), Kit expression levels were markedly decreased in cells overexpressing Lyn^WT^ (compare 663-Lyn^WT^ to 663-neo cells, Figure 5A). In contrast, EpoR expression was not affected. Next, we generated a vector encoding a dominant-negative form of Lyn (mutant Y397F) [22] tagged in its C-terminus (MT-Lyn^Y397F^) to inhibit Lyn function. Stable G418-resistant transfectants were selected and amplified in the presence of Epo. As illustrated in Figure 5B, expression of MT-Lyn^Y397F^ detected by immunoblotting with the MT antibody was associated with an increase in Kit level in the Epo-cultured cells. Thus, Lyn inactivation by a dominant-negative mutant allowed an up-regulation of Kit expression.

The biological consequences of Lyn overexpression were examined by studying cell proliferation over a 48 hrs period. Transfected cells continuously maintained in Epo before and during the selection process remained strictly Epo dependent since no growth occurred in the absence of Epo. At the three Epo concentrations tested, the number of living 663-Lyn^WT^ cells was approximately 2-fold increased compared to control 663-neo cells indicating that overexpression of Lyn was associated with a growth advantage (Figure 5C). In contrast to cells overexpressing Lyn^WT^ and grown with Epo, the growth of 663-Lyn^Y397F^ cells in the presence of Epo was reduced compared with control cells indicating that the dominant negative Lyn mutant was able to counteract the ability of Lyn to favor Epo-induced proliferation (Figure 5D). An opposite effect of Lyn was seen when cells were grown with SCF. Indeed, the growth rate of 663-Lyn^WT^ cells in the presence of SCF was reduced compared to 663-neo cells (Figure 5C). Thus, the intracellular presence of Lyn was not compatible with the expansion of proerythroblastic cells in the presence of SCF. This result is consistent with the ability of Lyn to down-regulate Kit expression.

## Discussion

During erythropoiesis, SCF and Epo tightly control the pool size of erythroid progenitor cells that will survive, divide or differentiate. From CFU-E to erythroblasts, the subtle balance between Epo and SCF responses results from a gradual decline of Kit expression associated to an increase in EpoR expression. However, the signaling mechanisms underlying the down-regulation of Kit and the up-regulation of EpoR are poorly understood, mainly because of the poor accessibility, the rarity and the transience of the CFU-E. The *spi-1* transgenic proerythroblasts no longer undergo differentiation and are arrested at the CFU-E/proerythroblast transition, but these cells retain their dependency to Epo or SCF for survival and proliferation. This experimental system, in which expansion is uncoupled from maturation, was used to examine the mechanisms controlling the proliferative responses of proerythroblasts to Epo or SCF. We show that Epo down-regulates Kit expression and that Lyn kinase behaves as a mediator of SCF signaling through controlling Kit expression.

The proerythroblastic cell lines derived from the spleen of diseased *spi-1* transgenic mice are morphologically [9] and cytologically (our unpublished data) similar, and resemble CFU-E/proerythroblastic cells. They have intrinsic proliferative capacities and express Kit and EpoR at different levels. This heterogeneity may reflect subtle differences in the level at which the maturation arrest provoked by the overexpression of the *spi-1* transgene occurred and/or to genetic or epigenetic variability in the cell lines.

We took advantage of this heterogeneity to investigate the molecular processes involved in the control of Kit and EpoR expression in response to their cognate ligands. Each cell line (six were studied and three are shown) had a proliferation rate in the presence of SCF that was correlated with the level of Kit expression. However, we observed that Kit levels were modulated by the cytokine used to expand the cells (Epo *versus* SCF) and that this modulation was rapidly reversible by a change in cytokine. Modifications in Kit levels were detected both in whole cell extracts and at the cell surface indicating that they *in fine* may lead to a modified response to SCF. The variations in surface expression of Kit analyzed by flow cytometry were significantly lower than that of total amount of cellular Kit analyzed by SDS-PAGE. These differences might be a consequence of the occurrence of Kit receptor internalization following SCF-induced activation [23], [24]. Because of the unavailability of EpoR antibodies for flow cytometric analysis, determination of the cell surface expression of EpoR was not performed. However, no obvious variation in EpoR expression could be detected in whole cell extracts whether cells were stimulated with Epo or SCF. The high Kit level in SCF-cultured cells was reminiscent of a mechanism involving SCF as a modulator of its own receptor expression as described for IL-3, CSF-1 and GM-CSF [25]. In contrast, we observed that Kit expression was down-regulated by Epo and that this down-regulation was inhibited by AG490, an inhibitor of EpoR signaling.

During hematopoiesis, Kit is expressed in immature and lineage progenitors and is down-regulated upon terminal erythroid differentiation. Accordingly, SCF enhances proliferation and retards differentiation of the erythroblasts and forced expression of Kit in erythroid precursors impairs their maturation [26], [27]. Different mechanisms leading to attenuation of Kit signaling have been reported. A mechanism involves SCF binding to Kit that induces a rapid internalization of the ligand-receptor complex and its degradation through an ubiquitination process [28], [29]. Another process refers to the down-regulation of Kit activation by negative effectors such as the SH2-containing protein tyrosine phosphatase SHP1 [30]. Such mechanisms can be ruled in our system since Kit down-regulation was seen in the absence of SCF, when cells were grown with Epo. Others mechanisms involve a regulatory control at the *Kit* transcriptional level. In erythropoiesis, transcription factors such as Tal-1 [31] and GATA-1 [27], [32] are repressors of *Kit* transcriptional expression. Our RT-PCR experiments revealed evident differences in *Kit* transcripts levels in cells cultured with Epo compared to cells cultured with SCF, a higher expression of *Kit* transcripts being detected in SCF-cultured cells. Though changes in mRNA stability are an alternate mechanism to explain a variation in mRNA amount, it is attempting to speculate that a transcriptional mechanism is most probably involved in the regulation of Kit expression in proerythroblastic cells. More investigations are required to characterize such a mechanism. Our findings of the down-regulation of Kit expression by Epo in the context of a late erythroid progenitor at the transition CFU-E/proerythroblast highlight that Epo is capable to switch off the expression of Kit in maturing erythroblasts. This process may also limit the cooperation between the two cytokines for proerythroblast proliferation and survival.

Several laboratories have described synergistic features between Kit and EpoR co-signaling to maintain the growth and survival of erythroid progenitors *in vitro* [33]–[37]. One mechanism involves EpoR as a direct downstream effector of Kit signaling through transphosphorylation induced by SCF. More indirectly, Kit signaling contributes to the sustained expression of Stat5 protein which can then be activated by Epo [38]. Target gene products of the EpoR-activated Stat5 axis can also contribute to enhance Kit signaling [39]. Finally, in the human hematopoietic stem cell-like cell line HML/SE, stimulation of Kit by SCF activates transcription of the *EpoR* gene making the cells responsive to Epo [40]. Similarly, we observed a cooperative effect between Epo and SCF for the survival and proliferation of *spi-1* transgenic proerythroblasts. It should be noted that cooperation was seen only at a limiting Epo concentration when Kit expression was up-regulated. In contrast, when Epo was used at a suboptimal concentration (1 U/mL) in combination with SCF, Epo was the only player in controlling cell proliferation. It is reasonable to propose that the down-regulation of Kit expression leads to the loss of SCF responsiveness. In this regard, SCF and Epo cooperation would be restricted to early stages of erythropoiesis when Kit level is high. This agrees with data localizing the physical interaction of Kit and EpoR before or at the CFU-E stage [34] and with results indicating that elevated levels of Epo abolish the requirement for SCF-mediated signals in *in vivo* erythropoiesis [41].

Our findings add to our knowledge of Lyn action in erythropoiesis. Analyses of Lyn-deficient mice revealed that CFU-E exhibited a reduced proliferative capacity associated with attenuated responses to Epo and SCF and that erythroblasts presented defects in survival and maturation [42]–[44]. It was thus proposed that Lyn was involved in the expansion of late erythroid progenitors and the development of mature erythroblasts. Lyn has been described as a downstream effector of Epo in promoting erythroid differentiation [22], [45]. Then, Lyn participates in Stat5 activation and phosphorylation of EpoR [46]. Our findings show that Lyn sustains the Epo-dependent proliferation agreeing with a role of Lyn in the proliferation of late erythroid precursor cells. Furthermore, ectopic Lyn overexpression in SCF-cultured cells resulted in a reduction in Kit expression making cells poorly responsive to SCF. This observation underlines a dual role for Lyn as a positive effector in Epo signaling and a negative effector in Kit signaling and presents Lyn as a major mediator of the balance between Epo and SCF responsiveness during proliferation of proerythroblastic cells.

Stat5 phosphorylation is induced by Epo in erythroid cells [19], [47] and this activation depends on kinases associated with EpoR: the Jak2 kinase [48] and tyrosine kinases of the Src family such as Lyn in murine [22], [46] and Src in human erythroblasts [49], [50]. Although the activation of Stat5 downstream of SCF/Kit has been reported in the myeloid MO7e cells [51], it remains infrequent. Indeed, Stat5 is not activated by SCF in the erythroid cell lines HCD57 [35] and G1E-ER2 [38] as well as in primary erythroid progenitors [52], [53], [54]. Similarly, we found that Stat5 was not activated by SCF in *spi-1* transgenic proerythroblasts. In contrast, Stat5 was activated by Epo. Because phosphorylated Stat5 levels in cells cultured under Epo paralleled the expression level of Lyn, a prediction could be that Stat5 was an effector downstream of Lyn in Epo-dependent proliferation of cells. Though Stat5 has been characterized as a direct substrat for Lyn during Epo-induced differentiation of the J2E cell line [22], it is not determined whether Lyn induces similar signaling during cell proliferation in response to Epo. Alternatively, Lyn was an Epo-responsive gene and could be a Stat5 transcriptional target gene. Induction of Lyn protein expression by Epo was associated to induction of RNA expression. Further investigation is required to know whether Stat5 controls the transcription of Lyn through direct binding to its transcriptional promoter.

In conclusion, our findings on the down-regulation of Kit expression by the EpoR/Lyn pathway in the context of a late erythroid progenitor cell (CFU-E/proerythroblast transition) provide some insights into the mechanisms leading to Kit extinction in maturing erythroblasts. We postulate that the control of Kit expression during erythroid development results from interference between Epo and SCF signaling where activation of the EpoR/Lyn pathway would lead the proerythroblast to the strict Epo dependency through mediating extinction of Kit expression. In this light, it will be interesting to determine if such a down-regulation of the activity of a cytokine active on early multipotent progenitors by a cytokine active later on the maturation of a lineage specific progenitor illustrates a general process in the hematopoietic development.
